# Notes and News

**Published:** 1899-12-23

**Authors:** 


### Notes and News.

(Collected and Communicated.)

An association of past and present resident officers of the Queen's Hospital, Birmingham, has been formed; and Sir James Sawyer has been elected as first president.

The pass lists for the recent examination for Bachelor of Surgery just issued by the University of London show that half of those in the first division are ladies from the Royal Free Hospital.

Provost Mackenzie, of Paisley, has opened the Royal Victoria Eye Hospital in that town, which represents his Jubilee offering to the district. Mr. Daniel Mackenzie, brother of the Provost, has endowed the institution with £1,000.

An interesting paper on "The Climatic Conditions Necessary for the Propagation and Spread of the Plague" was read before the Royal Meteorological Society on Wednesday by Mr. Baldwin Latham, Inst.C.E.

A cottage hospital has been opened at Skipton to serve the locality by Lady Frederick Cavendish. It has been erected as a Jubilee Memorial. There are male and female wards, each with six beds, a private ward for two paying patients with a sitting-room attached, an operating-room, and the usual offices. The amount subscribed for the purpose of establishing the memorial was about £5,500.

The new pathological buildings at the London Hospital are to serve as a memorial to the late Sir Andrew Clarke. The buildings, with the post-mortem rooms, chapel, and mortuary, are estimated to cost not less than £15,000 when completed. The London Hospital is being rendered efficient in every respect, and the expenditure upon absolutely necessary buildings to effect the result will not amount to less than £250,000.

The ridiculous position into which the anti-vaccinists have led the Leicester Board of Guardians seems to be having the effect of awakening people in other parts of the country to the absurdity of allowing themselves to be used by this discredited party for the furtherance of their crusade against the law. The Lynn Board of Guardians have now struck their colours, and by 13 votes to eight have decided to appoint a vaccination officer, a thing which they had before by a large majority refused to do.

A new wing has been opened at the Children's Hospital, Dublin, by the Countess of Cadogan.

Mr. J. S. Wood has given a donation of £100 to name a bed "In Memoriam" in the Chelsea Hospital for Women; and the Council, being in urgent need of funds, suggest the naming of beds in memory of those who have fallen in battle in South Africa.

The Victoria Hospital, Dundee, has been opened. It will contain 32 patients. The hospital was a mansion, and has been carefully adapted to institutional purposes. In the main building two wards are situated on the ground floor, and on the floor above are smaller wards. In the east wing is a ward for cancer patients. Attention has been paid to the comfort of the staff, for whom good accommodation has been provided.

We learn that the authorities of the Porthcawl Rest, near Cardiff, have been approached by the War Office as to the amount of accommodation which can be set aside for the sick and wounded on their return from Africa. The Cardiff Infirmary has offered 50 beds for the same purpose. The County Council will offer accommodation to the War Office, and private persons are most anxious to do the same. If the wounded are not well housed it will not be for the want of proffered accommodation.

The prize of 100 guineas offered by the Royal College of Surgeons, Edinburgh, for an original unpublished essay on a subject in any branch of surgery, or in anatomy, physiology, therapeutics, or pathology, bearing on surgery, open to all Fellows and Licentiates of the College other than members of the President's Council, has been awarded, from amongst the competitors, to Dr. Arthur Logan Turner, F.R.C.S.E., 20, Coates Crescent, Edinburgh, the title of whose essay was "Racial Characteristics of the Frontal Sinuses."

We have received the announcement of a winter course of entertainments for the in-patients of the National Hospital for the Paralysed and Epileptic. The programme which has reached us contains a notice to visitors which we consider worthy of note. It is to the effect that the wards will not be open to visitors at the conclusion of the entertainment. The regulation is a wise one under all similar circumstances; for, whilst there can be no doubt that a hospital is benefited by the intelligent interest taken in it by the public, a large influx of visitors to the wards, present more or less through aimless curiosity, is more harmful to the patients than beneficial to the financing of the hospital.

A short way with smoke producers is to make it illegal to burn coal which produces smoke. This is what has been done in certain parts of New York State, where, according to the New York *Medical News*, it has been held by the Courts that it is well within the police power of the State for the Legislature to declare that the burning of soft coal within certain prescribed limits of the city is detrimental to the public welfare, and should be prohibited. This is what has been done. Unfortunately for us most of the fuel used in this country is what in America is called soft coal. Still, we have plenty of hard coal, which we export in large quantities, to be used by people less enamoured of murky skies than we seem to be.

